# An illuminated respiratory activity monitoring system identifies priming-active compounds in plant seedlings

**DOI:** 10.1186/s12870-021-03100-8

**Published:** 2021-07-05

**Authors:** Judith Loogen, André Müller, Arne Balzer, Sophie Weber, Kathrin Schmitz, Roxanne Krug, Ulrich Schaffrath, Jörg Pietruszk, Uwe Conrath, Jochen Büchs

**Affiliations:** 1 AVT.BioVT, RWTH Aachen University, Forckenbeckstraße 51, 52074 Aachen, Germany; 2 Bioeconomy Science Center (BioSC), C/O Research Center Jülich, 52425 Jülich, Germany; 3 Department of Plant Physiology, RWTH Aachen University, Worringer Weg 1, 52074 Aachen, Germany; 4 Institute for Bio- and Geoscience, IBG-2: Plant Science, Forschungszentrum Jülich, 52425 Jülich, Germany; 5 Institut Für Bioorganische Chemie (IBOC), Heinrich-Heine-Universität Düsseldorf Im Forschungszentrum Jülich, 52426 Jülich, Germany; 6 Institut Für Bio- Und Geowissenschaften, IBG-1: Biotechnologie, Forschungszentrum Jülich, 52425 Jülich, Germany

**Keywords:** Sustainable agriculture, Oxygen transfer rate, Respiratory activity, Defense priming-inducing chemistry, Plant protection, Plant immunity

## Abstract

**Background:**

Growing large crop monocultures and heavily using pesticides enhances the evolution of pesticide-insensitive pests and pathogens. To reduce pesticide use in crop cultivation, the application of priming-active compounds (PrimACs) is a welcome alternative. PrimACs strengthen the plant immune system and could thus help to protect plants with lower amounts of pesticides. PrimACs can be identified, for example, by their capacity to enhance the respiratory activity of parsley cells in culture as determined by the oxygen transfer rate (OTR) using the respiration activity monitoring system (RAMOS) or its miniaturized version, µRAMOS. The latter was designed for with suspensions of bacteria and yeast cells in microtiter plates (MTPs). So far, RAMOS or µRAMOS have not been applied to adult plants or seedlings, which would overcome the limitation of (µ)RAMOS to plant suspension cell cultures.

**Results:**

In this work, we introduce a modified µRAMOS for analysis of plant seedlings. The novel device allows illuminating the seedlings and records the respiratory activity in each well of a 48-well MTP. To validate the suitability of the setup for identifying novel PrimAC in *Arabidopsis thaliana*, seedlings were grown in MTP for seven days and treated with the known PrimAC salicylic acid (SA; positive control) and the PrimAC candidate methyl 1-(3,4-dihydroxyphenyl)-2-oxocyclopentane-1-carboxylate (Tyr020). Twenty-eight h after treatment, the seedlings were elicited with flg22, a 22-amino acid peptide of bacterial flagellin. Upon elicitation, the respiratory activity was monitored. The evaluation of the OTR course reveals Tyr020 as a likely PrimAC. The priming-inducing activity of Tyr020 was confirmed using molecular biological analyses in *A. thaliana* seedlings.

**Conclusion:**

We disclose the suitability of µRAMOS for identifying PrimACs in plant seedlings. The difference in OTR during a night period between primed and unprimed plants was distinguishable after elicitation with flg22. Thus, it has been shown that the µRAMOS device can be used for a reliable screening for PrimACs in plant seedlings.

## Background

As the world population continuously grows, there is an increased need for food, feed, fibers, and bioenergy [29]. These stocks are mainly derived from crops, such as corn, rice or wheat, that are usually grown in large monocultures. These are heavily sensitive to biotic and abiotic stress. Synthetic pesticides are widely used to effectively fight pests and pathogens. However, they can accumulate in the soil or plant and, thus, they raise ecological and health concerns. In addition, the heavy use of pesticides provokes the emergence of pests and pathogens with insensitivities to the chemicals [29]. To avoid the risks that comes with synthetic pesticides, exploiting the plant immune system emerged as a supportive, or even alternative, approach for eco-friendly plant protection [8].

Upon attack by pathogens or after treatment with certain chemicals, plants can develop resistance or enhance their resistance to further pathogen attack [3]. This phenomenon is referred to as induced resistance which is frequently associated with defense priming [8]. The term refers to the enhanced capacity of plant cells to activate defense responses. Priming-associated induced resistance responses include systemic acquired resistance (SAR) [11]. SAR is a broad-spectrum immune response induced by salicylic acid (SA) and N-hydroxypipecolic acid [15, 33]. Thus, upon treatment with N-hydroxypipecolic acid, SA or substances with a similar mode of action, plant cells are frequently primed for the enhanced activation of defense responses [20, 42], and this usually comes with the establishment of SAR [8, 11]. Defense priming, whether induced by SA, treatment with another chemical compound, or previous pathogen attack frequently enhances the capacity of the plant also to ward off abiotic stresses, such as salinity, drought, flooding, chilling, or heat [18, 34, 41]. Until now, one common method to identify PrimACs is measuring the accumulation of mRNA transcripts of *WRKY6* and *WRKY53* [19]. *WRKY6* and *WRKY53* are reliable marker genes for defense priming in *A. thaliana* [19]. The accumulation of mRNA transcripts of these genes is not much induced during priming [19]. However, the accumulation of *WRKY6/53* transcripts induced by low doses of flg22 is more robust in primed than unprimed plants [19]. It has been demonstrated that priming involves considerably fewer costs and is hardly prone to pest or pathogen adaptation [26, 44]. Thus, triggering priming by eco-friendly chemicals represents a promising means for sustainable pest and disease control. However, identifying priming-inducing chemistry is difficult and applicable screening systems for priming activity are rare. Conrath and associates developed [20] and recently optimized [36] a mid- throughput screen for compounds that prime microbial pattern-induced furanocoumarin secretion in parsley cell suspension cultures. In addition, using a respiratory activity monitoring system (RAMOS), Schilling et al. [37] showed that their property can identify priming-active compounds (PrimACs) to enhance the respiratory activity, as measured by the oxygen transfer rate (OTR) of a parsley cell culture [1, 2]. In this study, the word respiration comprises the sum of the physical and biochemical processes in an organism by which oxygen is conveyed to tissues and cells. Two possible explanations for the enhanced oxygen consumption during priming have been previously discussed. One explanation claimed that the activation of mitochondrial alternative oxidase (AOX) might cause enhanced oxygen consumption [13, 14, 28, 32]. The other explanation suggested that the NADPH oxidase caused the enhanced oxygen consumption by the synthesis of reactive oxygen species [6, 13, 21]. Both explanations are conceivable separately or in combination.

For enhanced throughput, a µRAMOS device has been developed that utilizes parley suspension cells in 48-well microtiter plates (MTPs) [16]. The RAMOS technology repeatedly switches between phases of gassed and sealed bioreactors applying specific valves. During the sealed phase, the oxygen partial pressure, measured via a respective sensor, decreases due to respiration of the cultivated organisms [1]. The OTR from the gas to the aqueous phase is proportional to the determined slope of the oxygen partial pressure during the sealed phases [1, 16]. The μRAMOS is based on standard 48-well MTPs. It uses the same measurement principle as the RAMOS technology but is provided with another valve and sensor technology [16]. Whilst the RAMOS is equipped with electrochemical sensors, the µRAMOS uses immobilized fluorescence sensor spots to measure the oxygen partial pressure [16].

Both RAMOS and µRAMOS have been optimized for use with parsley cells in culture [16, 38, 40]. To overcome the limitations of (µ)RAMOS to parsley cell suspensions, in this work, a protocol for the use of intact plants in the µRAMOS device is developed. With the experimental setup, a light-emitting diode (LED) module is introduced that enables each well of a 48-well MTP to be individually irradiated with white light to ensure synchronized growth [5]. In this study, we present the setup for a LED-µRAMOS combination and a standard procedure for the identification of new PrimACs on plant seedlings. For a proof-of-principle of the introduced LED-µRAMOS combination and the standard procedure, a model screening for PrimACs has been performed using the newly synthesized methyl 1-(3,4-dihydroxyphenyl)-2-oxocyclopentane-1-carboxylate (Tyr020) as model test compound, which has been introduced by Krug et al. [23]. The power of the novel devise has been shown using SA as a positive control and spotting Tyr020 as a novel PrimAC in *A. thaliana*. Tyr020 was validated as a PrimAC using molecular biological analyses [10].

## Results

### LED module—µRAMOS combination

**Fig. 1:**
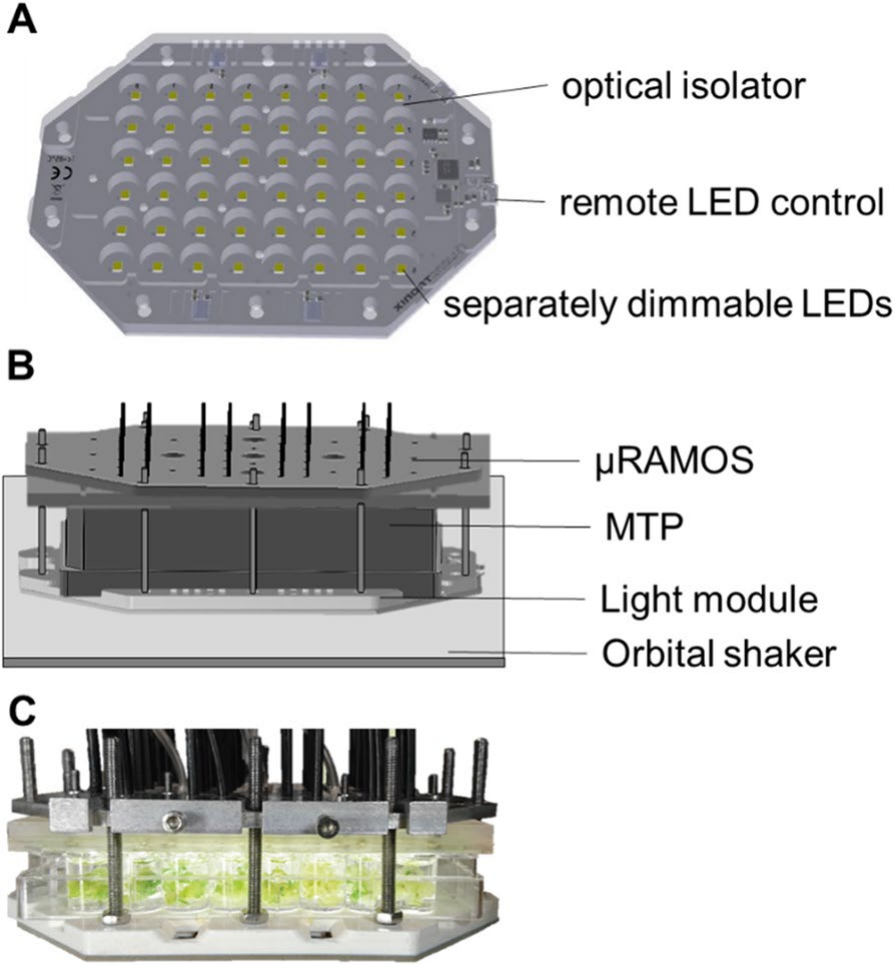
**A** Custom-made LED module. 48 individually dimmable LEDs for each well of a MTP. An optical isolator prevents interference between individual wells of the MTP. **B** Combination of the LED module with a μRAMOS device. The LED module is mounted below the MTP. μRAMOS measures the partial pressure of oxygen in each well and calculates the resulting OTR. **C** Photograph of the assembled device with *A. thaliana* plant seedlings. Translucent MTPs were used for illustration purposes. For online monitoring, black MTPs with transparent bottoms were used

A LED module was developed to enable the simultaneous growth of seeds. It enables adjusted light condition for germination and growth if needed, as it is the case for *A. thaliana* seedlings*.* The plant seedlings are grown in each well of a 48-well MTP in controlled light condition. The module consists of 48 individually dimmable LEDs (Lumitronix) mounted on a plate of the size of a commercial 48-well MTP (Fig. 1A). Optical isolators ensure that the light cannot enter neighboring wells, provided a black, clear-bottomed MTP is applied on top of the LED module. Because all wells are irradiated by an individual LED, equal conditions are maintained for each well, regardless of the well position. The µRAMOS device is applied atop the MTP (Fig. 1B) and enables a measurement of the respiration activity in each plate well. Figure 1C displays a photograph of the setup with *A. thaliana* seedlings on top of the LED module with the attached µRAMOS device. For illustration purposes, a translucent MTP has been drawn in this. This newly introduced combination of LED module and µRAMOS (LED-µRAMOS) is the basis of the presented protocol to identify new PrimACs.

### Standard procedure to screen for PrimACs in plants using µRAMOS

**Fig. 2:**
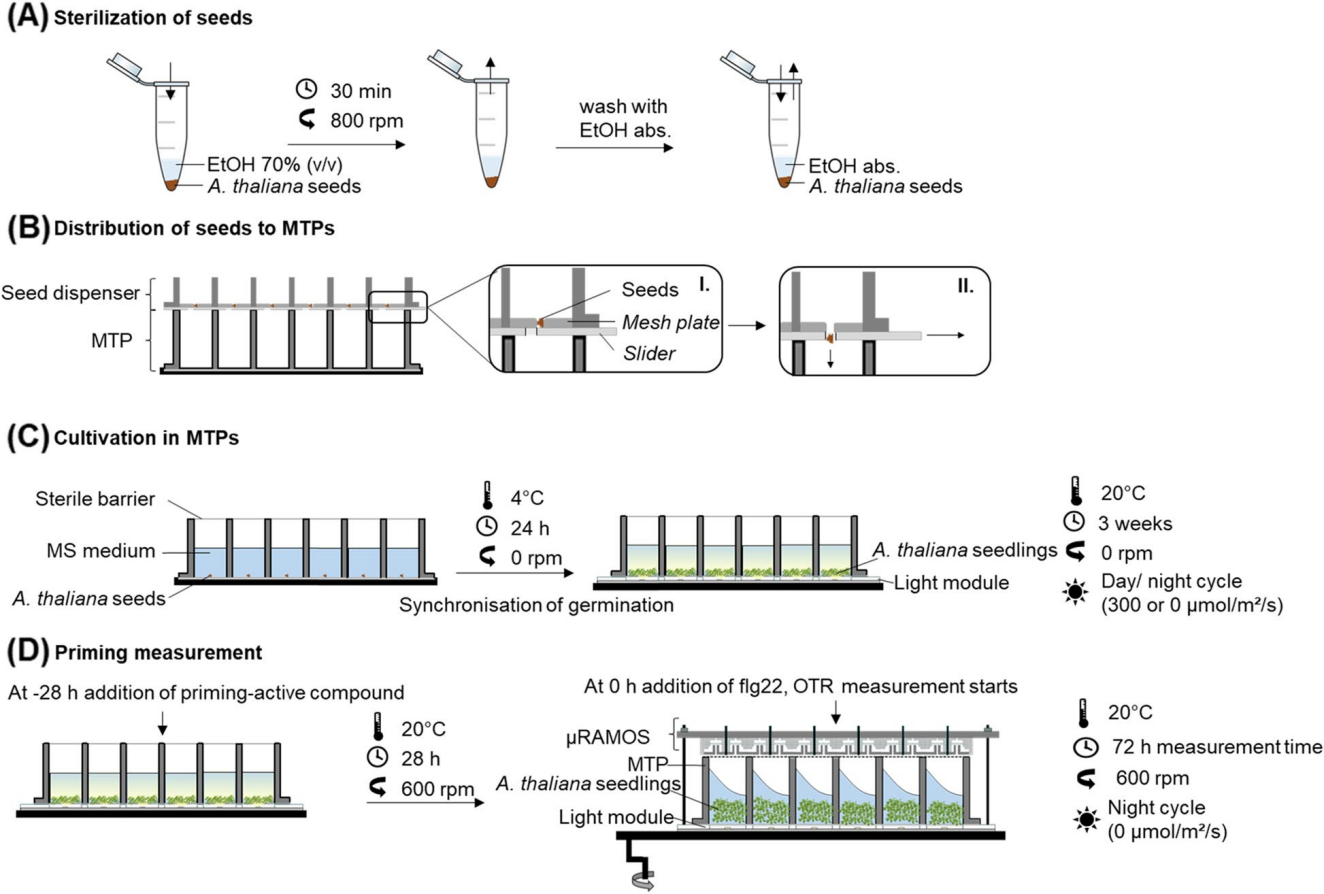
Scheme of the setup for PrimAC identification using *A. thaliana* seedlings in the LED-µRAMOS device. **A** Seeds were sterilized by washing with 70% (v/v) ethanol and ethanol absolute (EtOH abs.). **B** A seed dispenser was used to transfer sterilized seeds to the wells of a MTP. **C** Cultivation in MTPs for the effective and synchronized germination of seeds. *A. thaliana* was grown on 1.5 mL MS medium for 3 weeks at a 16-h day/8-h night cycle. **D** The respiratory activity was measured in the absence of light at 20 °C and 600 rpm shaking frequency with a shaking diameter of 3 mm. Treatments with compounds was done 28 h before the OTR measurement were started. Seedlings were elicited using 50 pM flg22 at time zero (0 h)

We developed a standard procedure that enables the search for PrimACs in the introduced LED-µRAMOS combination (Fig. 2). *A.* *thaliana* seeds were surface sterilized with 70% (v/v) ethanol (Fig. 2A). Using a seed dispenser, sterilized seeds were individually transferred into the wells of an MTP (Fig. 2B). Sterile MS medium was added to each well (Fig. 2C). To ensure synchronous and effective germination of seeds, the MTP was kept for 24 h at 4 °C. Then, the MTPs were incubated for 3 weeks at 20 °C. During incubation, a 16 h day/ 8 h night cycle was applied using the LED module (Fig. 2C). After those 3 weeks, the seedlings were treated with SA or Tyr020 (Fig. 2D) and incubated for another 28 h in growth condition. Then, seedlings were sprayed with 50 pM flg22. After elicitation, the respiratory activity of the seedlings was measured for 45 h by applying the µRAMOS (Fig. 2D) atop of the MTP.

### OTR of *A. thaliana* seedlings grown in the LED-µRAMOS combination

**Fig. 3:**
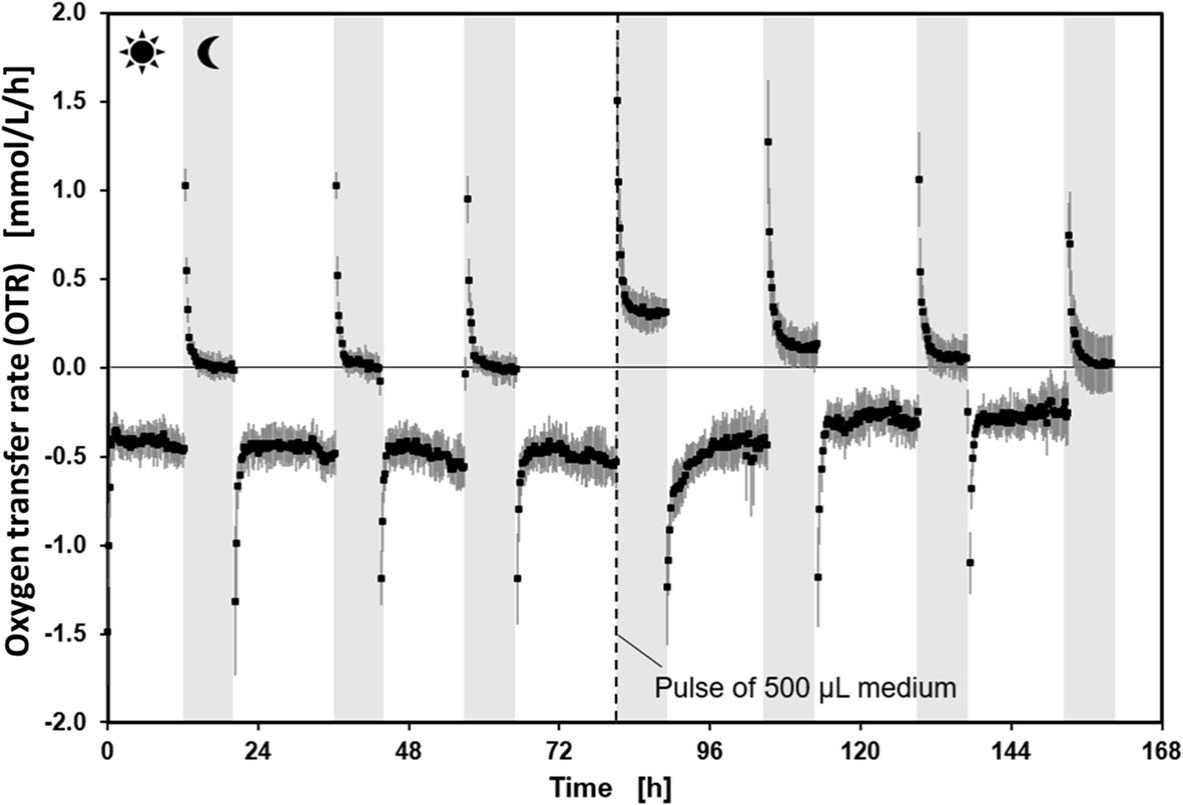
OTR of *A. thaliana* seedlings during 16 h day/8 h night cycle in the LED-μRAMOS combination. The white and grey background illustrate day and night periods, respectively, and are indicated by sun or moon. Three-week old *A. thaliana* seedlings, grown as in Fig. 2C, were transferred to the LED-μRAMOS combination and kept at 600 rpm shaking frequency, 3 mm shaking diameter, 20 °C, in 1.5 mL MS medium with 2.5 g/L sucrose, initial pH 5.7, light module irradiation intensity 300 µmol/m^2^/s at day time, 0 µmol/m^2^/s at night time. The standard deviation of 12 replicates is represented by the grey shadow around the data points

To validate the suitability of the novel LED-µRAMOS device for measuring the respiratory activity of *A. thaliana* seedlings, seedlings were grown in a MTP in the above described conditions in the LED-µRAMOS devise for 168 h. The OTR was monitored during the 16 h day/8 h night period. The course of the OTR over the 168 h period revealed that during the day, oxygen was produced as indicated by negative OTR values (Fig. 3). During the night, low amounts of oxygen were consumed, as obvious by positive OTR values (Fig. 3). However, the quantities of oxygen consumed were rather low, as obvious by the OTR being close to 0 mmol/L/h for most of the time in the night period, indicating starvation. At the end of the fourth day period, 500 µL of sterile MS medium were added to each well to avoid desiccation of the seedlings. This stimulated increased oxygen consumption during the following night period, as seen in Fig. 3 at 80 h. After supplementation with MS medium, the oxygen produced during the following day periods was lower than at the day periods before. Based on these results, it could be recognized that 2.5 g/L sucrose is not sufficient for a measurement to identify PrimACs. Thus, the MS medium was supplemented with 20 g/L sucrose to ensure that the seedlings are not starving. With this change, it is secured that the OTR remains above 0 mmol/L/h for an elongated night period, which is applied for the following PrimAC identification measurement. Positive OTR values are necessary to measure the influence of PrimACs on the respiration activity of the seedlings.

### Measurement of priming-inducting activity

**Fig. 4:**
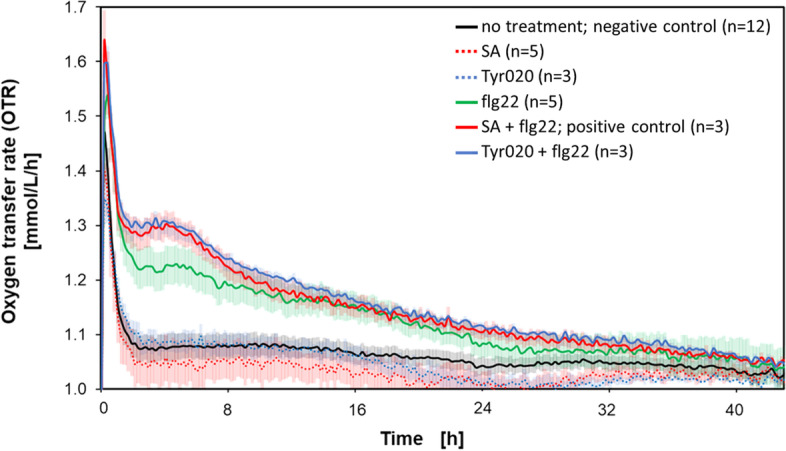
Respiratory activity of *A. thaliana* seedlings after treatment with priming compounds with and without flg22 elicitation, and reference cultivation without treatment with chemicals. OTR as a function of time of *A. thaliana* seedlings primed with 100 μM SA and 50 pM flg22 (red) or 25 µM Tyr020 and 50 pM flg22 (blue). Reference cultivations: without additives, negative control (black), addition of 100 μM SA only (red dotted line), 25 µM Tyr020 (blue dotted line), and with the addition of 50 pM flg22 only (green line). SA and Tyr020 were added at the -28 h time point relative to the time point of flg22 addition (0 h). Three-week-old *A. thaliana* seedlings were transferred to the µRAMOS device, according to Fig. 2D, and cultivated at 600 rpm shaking frequency, 3 mm shaking diameter, filling volume 1.5 mL, 20 °C, in MS medium with 20 g/L sucrose, initial pH 5.7. The number of replica (n) is specified for each condition in the legend. The standard deviation of replicates is indicated by the shadow surrounding the data

The screening for PrimACs was performed as illustrated in Fig. 2. *A. thaliana* seedlings were diveded in six groups and, where appropriate, treated at -28 h before elicitation with flg22 at time point 0 h. Immediately after elicitation, the OTR measurement was started (Fig. 4). In the first hour of recording, all measurements show significantly increased OTR values. These are various running-in phenomena of the µRAMOS device, for example, the MTP needs to first reach the temperature of the system before a stable OTR value can be measured. This period of the first hour is, therefore, irrelevant for the screening of PrimACs and is not further discussed. Twelve wells of the MTP were neither pretreated nor elicited to obtain a negative control (Fig. 4). The course of the OTR of the negative control remained at a low level (~ 1 mmol/L/h). Seedlings in 5 wells were treated with 100 µM SA and seedlings in 3 wells supplemented with 25 µM Tyr020 at -28 h. These 8 wells were not supplied with 50 pM flg22. This we did to record the course of the OTR of pretreated but unelicited plants (Fig. 4). The OTR curve of this group remained at ~ 1 mmol/L/h, comparable to the negative control.

 The course of the OTR (red and blue dotted) curves of these seedlings were like those of the negative control (black graph, Fig. 4). Furthermore, seedlings in 5 wells were not pretreated but elicited with 50 pM flg22 (green curve). After elicitation for 8 h, the OTR of the seedlings was significantly higher than that of the negative control. The OTR of the unprimed but elicited seedlings remained high for 40 h. Seedlings in 3 wells were treated with 100 µM SA (red curve, positive control) or 25 µM Tyr020 (blue curve) at -28 h before the OTR measurement was being started. These seedlings were elicited with 50 pM flg22 at the 0 h time point. The OTR values of SA-pretreated and later flg22-elicited seedlings were much higher than those of unprimed but elicited seedlings. The course of the OTR of Tyr020-treated and elicited seedlings was like the course of the OTR of the positive control. Thus, Tyr020 was identified as promising PrimAC candidate in *A. thaliana*.

**Fig. 5:**
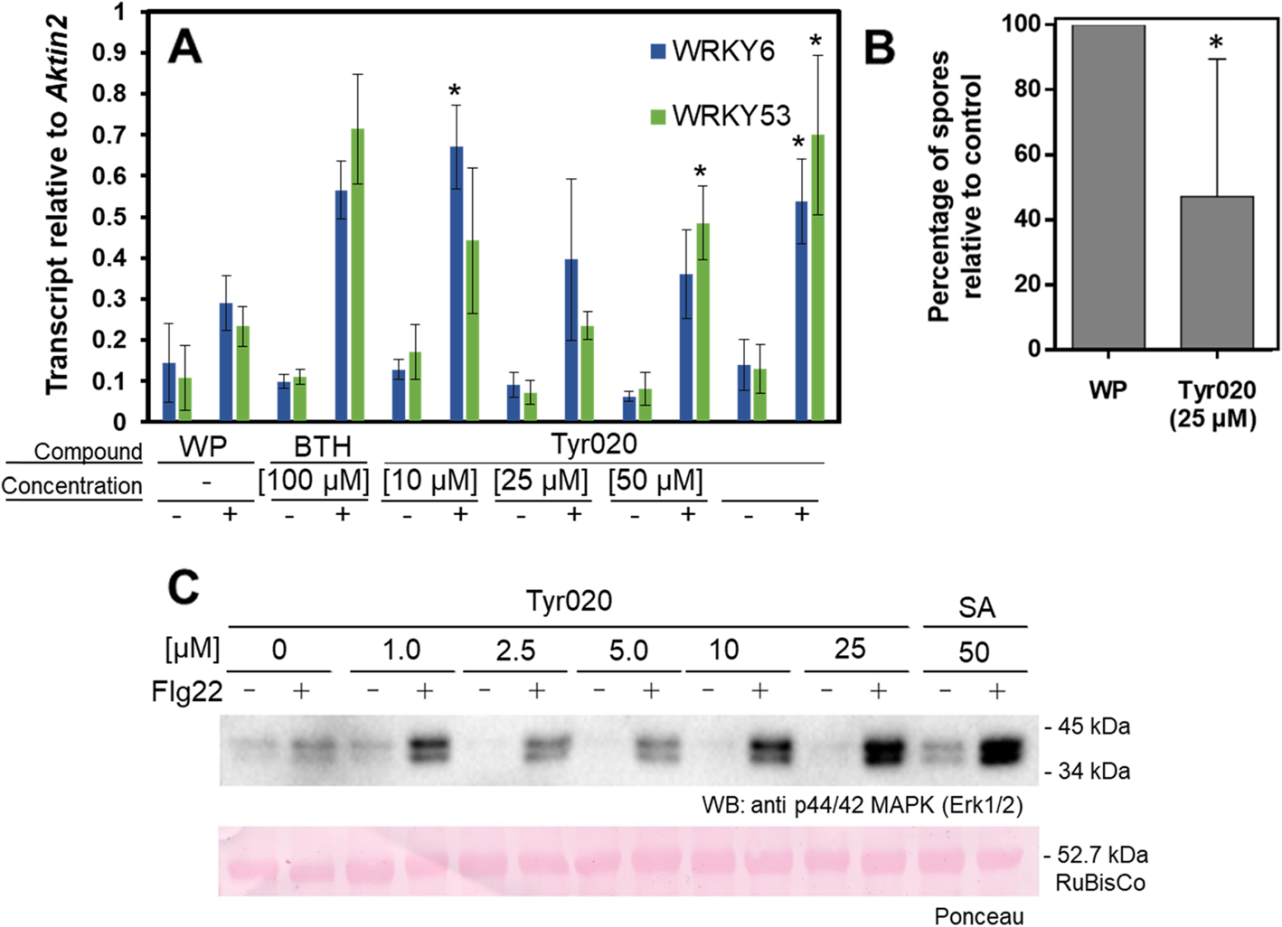
Verification of identified priming compound in soil grown *A. thaliana* plants. **A** Tyr020 primes *A. thaliana* plants for enhanced *WRKY6* and *WRKY53* defense gene activation. Six-week-old plants remained untreated (-) or sprayed with wettable powder ( +) or the indicated concentration of Tyr020 in wettable powder (WP) ( +) before they were elicited ( +) or not (-) with flg22. As positive control 100 µM Benzothiadiozole (BTH) was used. The accumulation of *WRKY6* and *WRKY53* mRNA transcript was determined by qRT-PCR. Stars (*) indicate significant differences between control (WP; +) and sample (*p* ≤ 0.05). **B** Sulforaphane (SFN) reduces downy mildew disease. Plants were treated with WP or a WP formulation of Tyr020 (25 µM). Twenty-four hours later, we spray-inoculated plants with a suspension of *Hyaloperonospora arabidopsidis* (Hpa) conidiospores (4 × 10^4^ spores per mL of water). Inoculated plants were kept at high humidity in short day. After 7 d, we determined the number of spores released by Hpa. Data were analyzed by Student’s *t* test. Asterisk denotes a statistically significant difference with 95% confidence. Data presented are means ± SD. *n* > 5. **C** *A. thaliana* seedlings pretreated with Tyr020 have enhanced phosphorylation of mitogen-activated protein kinase (MPK) activation motifs upon flg22 challenge. This indicates stronger MPK activation in Tyr020-pretreated plants. As positive control *A. thaliana* seedlings were treated with 50 µM salicylic acid (SA)

To verify or disprove Tyr020 as a PrimAC, we investigated whether the compound would induce known molecular mechanisms of defense priming in *A. thaliana* [8, 9]. To do so, we determined the accumulation of mRNA transcript of the *WRKY6* and *WRKY53* transcription factor genes inappropriately treated plants. To investigate whether Tyr020 would prime *A. thaliana* for the enhanced accumulation of *WRKY6/53* mRNA transcript, soil-grown *A. thaliana* seedlings were divided into six groups, each of six plants. One group was treated with a wettable powder (WP; negative control) which serves as a wetting agent for S-methyl benzo[d][1,2,3]thiadiazole-7-carbothioate (BTH). The second group was treated with a WP formulation of BTH (positive control), which is a functional SA analog Friedrich et al. [15, 24] that strongly triggers defense priming in *A. thaliana* [19, 22]. The four remaining groups of seedlings were treated with different concentrations of Tyr020. Forty-eight hours after treatment, three seedlings of each group were elicited with flg22. The remaining three seedlings of each group remained unelicited. Three hours after flg22 elicitation, seedlings were harvested and analyzed for the accumulation of mRNA transcript of *WRKY6* and *WRKY53* by qRT-PCR. As shown in Fig. 5A, the flg22-elicited seedlings of the WP group showed a small increase in the accumulation of *WRKY6* and *WRKY53* mRNA transcript compared to the unelicited seedlings. The unelicited group of BTH-treated seedlings showed a similar extent of mRNA transcript accumulation of the two *WRKY* genes (Fig. 5). When compared to the WP group of seedlings, the flg22-elicited group of BTH-pretreated seedlings displayed a significantly higher accumulation of *WRKY6* and *WRKY53* transcript (Fig. 5A). This result confirms the defense priming-inducing activity of BTH in *A. thaliana* seedlings and verifies that the experimental setup allows the detection of priming-inducing activity. The Tyr020-treated but unelicited seedlings accumulated *WRKY6* and *WRKY53* mRNA transcript to an extent like the unelicited WP group (Fig. 5A). When compared to the WP groups, the Tyr020 pretreated and later elicited group of seedlings had an increased level of mRNA transcript of both these genes. Only at 100 µM Tyr020, pretreated plants showed a significant (*p* ≤ 0.05) increase in the accumulation of *WRKY6* and *WRKY53* mRNA transcript when compared to the WP group. The groups of seedlings that were treated with 10 or 50 µM Tyr020 only for one of the *WRKY* genes showed a significant increase in mRNA transcript levels (Fig. 5A). However, in sum, the results of this experiment disclose the priming-inducing capacity of Tyr020 at the level of defense gene expression, and they reveal that Tyr020 seems to poise *WRKY6/53* for enhanced transcription upon further challenge.

To investigate whether Tyr020 would affect the infection of *A. thaliana* by the oomycete pathogen *Hyaloperonospora arabidopsidis* (*Hpa*), *A. thaliana* (Col-0) plants were sprayed with a wettable powder (WP) formulation of Tyr020 before they were inoculated with *Hpa*. The Noco race of *Hpa* causes downy mildew disease on *A. thaliana* Col-0 plants (Coates and Beynon [12]). Figure 5B shows that pretreatment with Tyr020 seems to reduce the susceptibility of *A. thaliana* to downy mildew disease, as obvious by reduced *Hpa* sporulation on Tyr020-pretreated plants. This suggests that Tyr020 activates defense priming and reduces the susceptibility to *Hpa* infection in *A. thaliana*.

To further investigate Tyr020’s mode of action, the accumulation of phosphorylated mitogen-activated protein kinases (MPKs) was analyzed in *A. thaliana* seedlings upon adequate treatments. Beckers et al. [4] demonstrated that in this plant defense priming is associated with enhanced levels of latent MPK3 and MPK6. However, these enzymes remained catalytically inactive until further stimulation of the plants [4]. Once challenged, more MPK molecules became phosphorylation-activated in primed compared to unprimed plants [4]. To investigate the phosphorylation of MPKs in Tyr020-treated *A. thaliana* seedlings, appropriately treated seedlings were subjected to western blotting analysis and immunodetection with phospho-motif-specific antibodies (Fig. 5C). Two untreated seedlings were used as a negative control, whereas two other seedlings were treated with 50 µM SA (positive control) to activate defense priming. Ten seedlings were treated with Tyr020 at concentrations from 1 to 25 µM. One seedling of each condition was later elicited with flg22. As shown in Fig. 5C, MPK phosphorylation was detected in all seedlings elicited with flg22. For the unpretreated but elicited seedling, the phosphorylation signal was very low compared to the elicited positive control. No or only weak MPK phosphorylation was found in all the unelicited samples. Except for 1 µM, Tyr020 with increasing concentration strongly enhanced the flg22-induced MPK phosphorylation signal that, at 25 µM, was almost as strong as the phosphorylation signal in seedlings that were primed with 50 µM SA and later challenged with flg22 (Fig. 5C). This result supports the above conclusions from the µRAMOS and gene expression studies, claiming Tyr020 as a previously unknown PrimAC.

## Discussion

An LED module was developed that allows equal light exposure of plant seedlings in each well of a 48-well MTP. Development of a similar illumination device has been published previously [27]. The described resulting improvements of individually illuminated plant cultivation could be reproduced with the introduced LED module. The first measurement (Fig. 3) with the LED-µRAMOS combination showed that MS medium supplemented with 2.5 g/L sucrose was not sufficient to measure respiration activity of the seedlings during the night period, as indicated by OTR values close to 0 mmol/L/h. Schilling et al. have shown that PrimACs enhance the respiration activity of plant cells [37]. Therefore, for an application of the LED-µRAMOS combination for the successful identification of PrimACs, measurable respiration of the seedlings is required. The problem that due to starvation, no respiration activity was measured at night could be overcome by the addition of fresh MS medium. The addition of fresh medium to *A. thaliana* in the LED-µRAMOS leads to an increased OTR during the following night cycle, showing the increased metabolic activity of the plant seedlings due to the newly added nutrients. The MS medium supplemented with sucrose seems to satisfy the seedlings’ carbon demand and, therefore, the photorespiration might have become lower, as it was already published for sugarcane by Lobo et al. [25]. In the second and third night cycle after addition of fresh medium, the OTR was still higher than before addition. In summary, the addition of sterile medium was successful so that in the future, longer cultivation of plant seedlings is also conceivable. In order to reduce the risk of possible contamination by the addition of fresh medium and to minimize the handling steps, it was decided to increase the initial concentration of the supplemented sucrose from 2.5 g/L to 20 g/L.

This result suggested that metabolic changes of the plant seedlings seem to be more promoted during the night cycle. As previous studies on plant suspension cell cultures already showed, the respiration activity gives crucial information about their metabolism [17, 31, 43].

Hence, the evaluation of candidates of PrimACs is best executed during an elongated night period. The growth was performed applying a 8 h day/16 h night cycle, using the LED module. The beginning of the elongated night period was only associated with the addition of flg22. At the same time, the µRAMOS device was mounted on top of the MTP, and the OTR measurement started. After elicitation, primed plant seedlings showed much higher OTR values than unprimed, but elicited, or only elicited plant seedlings (Fig. 4). Thus, PrimAC enhance the capacity of respiratory activity of intact plants. This enhanced capacity can be measured with the LED-µRAMOS combination, but other oxygen-based measurement systems might also be conceivable. The measurement systems published by Scafaro et al. [35], or O'Leary et al. [30], both relying on the Q2 oxygen sensor (Astec Global, The Netherlands), measure the oxygen consumption of leaves and roots. Nevertheless, both measurement systems could potentially be used for PrimAC identification. As this enhanced capacity of respiratory activity is just measured at this stage of experimental protocol, the µRAMOS device needs to be applied, while the germination, growth and priming can be performed separately in a microtiter plate. Through time-shifted initiation of cultivations in microtiter plates, the screening of 48 plant seedlings every 40 h per µRAMOS device becomes possible. The results of the model screening (Fig. 4) indicate that it might be possible to reduce this measurement period to 8 h since the difference between successfully primed plant seedlings and the corresponding negative control is most evident in the first 8 h after elicitation. A shortened measurement period would further enhance the throughput of the screening method to up to 48 plant seedlings per 8 h per µRAMOS device.

## Conclusions

In this paper, the use of the µRAMOS for intact plant seedlings in 48-well MTP was demonstrated. The major advantage of using intact plants rather than cell suspension cultures is the differentiation status and overcoming the limitations of parsley and some other plant species. µRAMOS is likely compatible with essentially any crop and, thus, closer to application. However, in contrast to soil-grown plants, measurements can be done at early growth stages in controlled conditions, as parameters like nutrition and light become adjustable. By switching from the RAMOS device, which allows simultaneous screenings in eight shake flasks, to the µRAMOS device, which enables 48 simultaneous screenings, the throughput per experiment was drastically increased. Based on the increased OTR after elicitation (Fig. 4), Tyr020 has been identified as previously unknown PrimAC.

The newly introduced LED-µRAMOS combination enables individual light intensities and irradiation periods for each well, while the OTR is independently measured online. This allows for different irradiation intensities, which might be required by different plant species within one experiment. In the future, candidates for PrimACs and combinations thereof could simultaneously be tested for their priming-inducing activity in different crops.

## Methods

### Chemicals

SA, BTH and wettable powder are commercially available and Tyr020 has been synthesized according to Krug et al. [23]. Krug et al. published this compound under the name compound 8a [23]. SA, BTH, Tyr020 and wettable powder were diluted in distilled water according to the indicated concentrations. No additional solvent was used.

### LED module

The LED module is custom-made by Lumitronix® and consists of 48 individual dimmable LEDs (Fig. 1). The plate can be mounted underneath a transluminescent-bottomed 48-well MTP resulting in one LED beneath each well. To allow a light gradient within the plate, each LED is individually adjustable. Optical isolators prevent stray light from LEDs into neighboring wells. The OTR was determined in shake flasks using the RAMOS [1, 2] in MTPs using the µRAMOS. Both devices were developed and built in house [16, 38]. Measurements were performed using black, clear-bottom microtiter plates. The µRAMOS device was mounted on top of the MTP. The measurement was performed at 20 °C with a shaking frequency of 600 rpm and a shaking diameter of 3 mm. The filling volume was 1.6 mL per well. The OTR was calculated based on measured partial pressure of oxygen and registered settings, including the headspace volume. This calculation was adjusted to changing headspace volumes, i.e., after the addition of PrimACs or fresh medium.

### *A. thaliana* growth

*Arabidopsis* seeds (*Arabidopsis thaliana*, accession Col-0) were purchased from the European Arabidopsis Stock Centre (Nottingham Arabidopsis Stock Centre; NASC). The seeds were washed in 70% (v/v) ethanol for 30 min and then twice in 100% (v/v) ethanol for 1 min, according to Fig. 2. Dried seeds were transferred to a MTP using a seed dispenser to a number of 15 ± 5 seeds per well. The seeds were provided with 1.5 mL (MS) medium. MS medium, including vitamins (M0222; Duchefa Biochemie B.V.), was prepared as recommended by the manufacturer and supplemented with 2.5 g/L sucrose. The pH value was adjusted to 5.7 using 0.01 M potassium hydroxide, before the medium was autoclaved (121 °C, 20 min).

For stratification the seed-loaded MTPs were stored at 4 °C overnight to ensure synchronous and efficient germination [5]. On the next days, the plates were cultivated on the LED module at 20 °C. After 3 weeks, the µRAMOS device was mounted atop.

### Cultivation, priming and elicitation of soil-grown *A. thaliana* seedlings

*A. thaliana* (accession Col-0; NASC) seedlings were grown on soil with a 16 h day/ 8 h night cycle applied in a pests-free room. When needed, the soil was irrigated. After 4 - 6 weeks, seedlings were used for conventional priming experiments. The leaves of *A. thaliana* seedlings were sprayed with Tyr020 dilutions in ascending concentrations between 1 µM and 100 µM. As positive control, the leaves of another plant seedling were sprayed with 100 µM BTH. As negative control, leaves of plant seedlings were sprayed with 21% (w/v) wettable powder in water. Every condition was applied on six plant seedlings each. The plant seedlings were further grown for 48 h, before three plants of each condition were infiltrated with 1 nM flg22 solution.

### Determination of *WRKY6*, *WRKY53* and *Actin*2 expression

Five hours after elicitation, leaves were harvested and homogenized. RNA was isolated from frozen leaves using the TRIZOL method, as published by [7]. The RT-qPCR reactions were performed in a 10 µL volume. The PCR mixture was consisting of 2.7 µL nuclease-free water, 5 µl Takyon TM No Rox SYBR MasterMix dTTP Blue (Eurogentec), 0.15 µL forward primer, 0.15 µL reverse primer, and 2.5 µL template. The according primers are listed in Table 1. The amplification was performed using a PCR machine (Applied Biosystems). The PCR was performed for 3 min at 95 °C as initial denaturation, followed by 40 cycles of 15 s at 95 °C for denaturation, 60 s at 60 °C as annealing, 15 s at 95 °C for extension. The final extension was set to 60 °C for 1 min.

**Table 1:** Primers used for RT-qPCR

Primer	Sequence 5´-3´
*WRKY6*	ACTTCACGGTCATTATCTCCAGC TGAATTTAGGTTTCCGGTGAGTC
*WRKY53*	CTCCATCGGCAAACTCTTCAC CCGAGCGTACAACTTATTCCG
*Actin2*	GGTAACATTGTGCTCAGTGGTGG GGTGCAACGACCTTAATCTTCAT

### SDS-PAGE, western-blotting analysis and immunodetection

To detect MPK phosphorylation sites, leaves of the soil-grown seedlings were harvested and homogenized at 5 h after elicitation. Frozen tissue was either ground using the Precellys 24 homogenizer (Bertin Instruments). All tubes and buffers were kept cold during the procedure. Ground plant material was washed twice with 100% acetone and centrifuged at 16,100 g at 4 °C for 5 min. The pellet was resuspended in 10% (w/v) TCA in acetone and were transferred to an ultrasound ice-water bath for 10 min followed by another centrifugation step (16,100 g, 4 °C, 5 min). Then, the pellet was washed once with 10% (w/v) TCA in acetone, once with 10% TCA (w/v) and once with 80% (v/v) acetone. The supernatant was discarded and the pellet was resuspended at RT in freshly prepared dense SDS buffer (100 mM Tris–HCl pH 8.0, 30% (w/v) sucrose, 2% (w/v) SDS, 5% (v/v) β-mercaptoethanol). An equal volume of phenol/Tris–HCl pH 8.0 (Applichem) was added to each tube. Samples were centrifuged at 16,100 g at RT for 20 min. The upper phase of each tube was split into two new tubes, 5 volumes of 100 mM ammonium acetate in methanol were added, and total protein was precipitated at -20 °C for 60 min. Proteins were collected by centrifugation at 16,100 g at 4 °C for 5 min. Pellets were washed once with 100 mM ammonium acetate in methanol and once with 80% (v/v) acetone, and the pellets were dried. To determine total protein concentration, pellets were resuspended in buffer (7.7 M urea, 2 M thiourea, 300 mM NaCl, 0.25% (w/v) CHAPS, 50 mM NaH_2_PO_4_ pH 8, 50 mM Tris pH 8, 20 mM imidazole) and incubated at RT for 1 h before Bradford protein assay (Quick-Start Bradford; BioRad) was performed. Protein samples were resuspended in loading buffer (10X Sample Reducing Agent, 4X LDS Sample Buffer; NuPAGE) to equal concentrations and heated at 95 °C for 10 min. The denatured samples were applied to a two parted polyacrylamide gel, consisting of a 4% collection gel and a 12% separation gel. The pockets were loaded with 5 µL sample, and gel electrophoresis was performed at 175 V for 60 min. MES, pH 7.3, was used as running buffer.

For the subsequent western blot, the proteins were electrophoretically transferred from the SDS gel to a nitrocellulosemembrane (Carl Roth) applying 60 min at 250 mA. After blotting, the membrane was washed 2 times for 5 min each in TBST buffer (20 mM tris; 150 mM NaCl; 0.1% (v/v) Tween 20). The blocking was performed for 60 min in a 5% (w/v) skimmed milk powder (Nutricia Protifar) in TBST buffer solution. After blocking, the membrane was washed 2 times for 5 min each in TBST buffer. The primary antibody anti p44/42 (Cell SignalingTechnologyR) was diluted 1:1000 in a 5% (w/v) Bovine Serum Albumin (Panreac AppliChem) in TBST buffer solution. The membrane was incubated for 12 h at 10 °C. After incubation, the membrane was washed twice for 5 min in TBST buffer.

As secondary antibody, the anti-rabbit IgG, HRP-linked Antibody #7074 (Cell SignalingTechnologyR) was applied. It was diluted 1:2000 in a solution of 5% (w/v) skimmed milk powder in TBST buffer solution. The membrane was incubated for 1 h at room temperature in the solution before being washed twice in TBST for each 5 min.

For protein detection, the ChemiDocTMMP Imagine System (BioRadR) with white light irradiation was used.

### Ponceau staining

To check for equal protein loading, the membrane was stained with Ponceau S. For staining, the Ponceau S solution (Panreac AppliChem) was used, and the producer’s protocol was followed precisely.

### Determining *A. thaliana* susceptibility to *Hyaloperonospora arabidopsidis* (Hpa)

*A. thaliana* Col-0 plants were grown in short-day (8 h light of 120 µmol/m^2^/s^1^, 22 °C, 65% relative humidity). Two-week-old plants were sprayed with wettable powder (WP) (control) or a WP formulation of Tyr020 (final concentration 25 µM). 24 h after treatment, plants were inoculated by spraying with a conidiospore suspension of Hpa (race Noco; 4 × 10^4^ spores per mL of water). Inoculated plants were covered with a transparent lid to ensure high humidity and kept in short-day condition. After 7 d, the number of spores released by Hpa was determined as described by Schmitz et al. [39].

## Data Availability

The datasets generated during and analyzed during the current study are available from the corresponding author on request.
